# E-Waste Management: As a Challenge to Public Health in India

**DOI:** 10.4103/0970-0218.69251

**Published:** 2010-07

**Authors:** Monika, Jugal Kishore

**Affiliations:** Department of Community Medicine, Maulana Azad Medical College, New Delhi, India

## Introduction

In India, the quantity of “e-waste” or electronic waste has now become a major problem. Disposal of e-waste is an emerging global environmental and public health issue, as this waste has become the most rapidly growing segment of the formal municipal waste stream in the world.(1) E-waste or Waste Electrical and Electronic Equipment (WEEE) are loosely discarded, surplus, obsolete, broken, electrical or electronic devices.(2) In India most of the waste electronic items are stored at households as people do not know how to discard them. This ever-increasing waste is very complex in nature and is also a rich source of metals such as gold, silver, and copper, which can be recovered and brought back into the production cycle. So e-waste trade and recycling alliances provide employment to many groups of people(3) in India. Around 25,000 workers including children are involved in crude dismantling units in Delhi alone where 10,000–20,000 tonnes of e-waste is handled every year by bare hands. Improper dismantling and processing of e-waste render it perilous to human health and our ecosystem. Therefore, the need of proper e-waste management has been realized.(4) It is necessary to review the public health risks and strategies to combat this growing menace.

## Burden of E-Waste

In India, solid waste management, with the emergence of e-waste, has become a complicated task. The total waste generated by obsolete or broken down electronic and electrical equipment was estimated to be 1,46,000 tonnes for the year 2005, which is expected to exceed 8,00,000 tonnes by 2012.(2) However, according to the Greenpeace Report, in 2007, India generated 380,000 tonnes of e-waste. Only 3% of this made it to the authorized recyclers’ facilities. One of the reasons for this is that the India has also become a dumping ground for many developed nations. The Basel Action Network (BAN) stated in a report that 50-80% of e-waste collected by the USA is exported to India, China, Pakistan, Taiwan, and a number of African countries.(5) India is one of the fastest growing economies of the world and the domestic demand for consumer durables has been skyrocketing. From 1998 to 2002, there was a 53.1% increase in the sales of domestic household appliances, both large and small all over the world.(6) Another report estimated that in India, business and individual households make approximately 1.38 million personal computers obsolete every year,(7) accelerating the rate of e-waste generation, which is around 10%, annually(8) going to affect environmental health indicators.(9)

## Health Impacts

Electronic equipments contain many hazardous metallic contaminants such as lead, cadmium, and beryllium and brominated flame-retardants [Table 1]. The fraction including iron, copper, aluminum, gold, and other metals in e-waste is over 60%, while plastics account for about 30% and the hazardous pollutants comprise only about 2.70%.(10) Of many toxic heavy metals, lead is the most widely used in electronic devices for various purposes, resulting in a variety of health hazards due to environmental contamination.(11) Lead enters biological systems via food, water, air, and soil. Children are particularly vulnerable to lead poisoning – more so than adults because they absorb more lead from their environment(12) and their nervous system and blood get affected. It is found that the e-waste recycling activities had contributed to the elevated blood lead levels in children living in China, which is one of the popular destinations of e-waste.(13) This was due to that fact that the processes and techniques used during the recycling activities were very primitive. Various studies have reported the soaring levels of toxic heavy metals and organic contaminants in samples of dust, soil, river sediment, surface water, and groundwater of Guiyu in China. In the same areas, the residents had a high incidence of skin damage, headaches, vertigo, nausea, chronic gastritis, and gastric and duodenal ulcers.(14) Further it was found that the blood lead levels of children were higher than the mean level in China, and there was no significant difference between boys and girls.(15)

**Table 1:** Various e-waste sources, their constituents, and health impacts(5)

E-waste sources	Constituents	Health effects
Solder in printed circuit boards, glass panels, and gaskets in computer monitors	Lead	Damage to central and peripheral nervous systems, blood systems, and kidney damage
		Adverse effects on brain development of children; causes damage to the circulatory system and kidney
Chip resistors and semi-conductors	Cadmium	Toxic irreversible effects on human health
		Accumulates in kidney and liver
		Causes neural damage
Relays and switches, and printed circuit boards	Mercury	Chronic damage to the brain
		Respiratory and skin disorders due to bioaccumulation in fishes
Galvanized steel plates and decorator or hardener for steel housing	Chromium	Causes bronchitis
Cabling and computer housing	Plastics and PVC	Burning produces dioxin that causes reproductive and developmental problems
Electronic equipment and circuit boards	Brominated flame-retardants	Disrupt endocrine system functions
Front panels of CRTs	Barium, phosphorus, and heavy metals	Cause muscle weakness and damage to heart, liver, and spleen
Copper wires, Printed circuit board tracks.	Copper	Stomach cramps, nausea, liver damage, or Wilson’s disease
Nickel–cadmium rechargeable batteries	Nickel	Allergy of the skin to nickel results in dermatitis while allergy of the lung to nickel results in asthma
Lithium-ion battery	Lithium	Lithium can pass into breast milk and may harm a nursing baby
		Inhalation of the substance may cause lung edema
Motherboard	Beryllium	Carcinogenic (lung cancer)
		Inhalation of fumes and dust causes chronic beryllium disease or beryllicosis

It was found that e-waste recycling operations were causing higher levels of polychlorinated dibenzo-*p*-dioxins and polychlorinated dibenzofurans (PCDD/Fs) in the environment as well as in humans. Body burdens of people in hair, human milk, and placenta from the e-waste processing site showed significantly higher levels of polychlorinated dibenzo-*p*-dioxins and polychlorinated dibenzofurans (PCDD/Fs) than those from the non-processing site.(16,17) There is paucity of data on burdens of heavy metal exposure on human body in India. A large number of workers including small children are exposed to different dismantling activities of e-waste. Although findings of these studies cannot be generalized to India but these are enough to alarm and strongly suggest to be replicating in occupational settings in India. There are no data available about the health implications of these workers. They might be ruining their lives in the lack of appropriate knowledge.

In another study from China, human scalp hair samples were collected to find out heavy metal exposure to workers from intense e-waste recycling sites. Higher concentrations of Pb, Cu, Mn, and Ba metals were found in hair of exposed as compared to the hair in control group.(18)

## Current Status of E-Waste Management

For the recycling of e-waste, India heavily depends on the unorganized sector as only a handful of organized e-waste recycling facilities are available. Over 95% of the e-waste is treated and processed in the majority of urban slums of the country, where untrained workers carry out the dangerous procedures without personal protective equipment, which are detrimental not only to their health but also to the environment.

Recycling and treatment facilities require a high initial investment, particularly those fitted with technologically advanced equipments and processes.(19) For the dismantling of one computer piece, these workers only get Rs. 5 or 10. For such a small amount, workers ruin their lives.(20) Such “backyard recyclers” do not have wastewater treatment facilities, exhaust-waste gas treatment, and personal health protection equipment.(21) Williams(22) observed that despite significant attention from the media and enactment of some national level trade bans (most notably, China and India), the problem is apparently worsening. Therefore, health risk assessments are also required for the analysis of the consequences and of inappropriate management of end-of-life electronic wastes in developing countries.(23)

## E-Waste Management Initiative

In Environmental (Protection) Act 1986, the “polluter pays principle” is enacted to make the party responsible for producing pollution responsible for paying for the damage done to the natural environment.(24) In international environmental law, it is mentioned in principle 16 of the Rio Declaration on Environment and Development. Polluter pays is also known as extended producer responsibility (EPR). Under the Environment (Protection) Act 1986, central and state governments can enact legislations to safeguard the environment and people from exposure to toxic and hazardous nature of waste. Any violation of the provision of this act or notified rules is liable for punishment.(25) Such penalty can be imposed on the violator if specific rules and regulations on e-waste are violated.

CPCB India is finalizing the set of rules and most recently issued a formal set of guidelines for proper and eco-friendly handling and disposal of the electronic waste. The Ministry of Environment and Forests is now processing the rules framed by electronics equipment manufacturers with the help of NGOs. According to the new guidelines issued by CPCB in 2007, e-waste is included in schedules 1, 2, and 3 of the “Hazardous Waste (Management and Handling) Rules 2003” and Municipal Solid Waste Management Rule, 2000.(2) Each manufacturer of a computer, music system, mobile phone, or any other electronic gadget will be “personally” responsible for the final safe disposal of the product when it becomes a piece of e-waste. Department of Information Technology (DIT),(26) Ministry of Communication and Information Technology, has also published and circulated a comprehensive technical guide on “Environmental Management for Information Technology Industry in India.” Demonstration projects have also been set-up by the DIT at the Indian Telephone Industries for the recovery of copper from Printed Circuit Boards.

As an effort to make the users aware of the recycling of e-waste, many electronic companies such as Apple, Dell, and HP have started various recycling schemes.(27) Nokia India announced its “recycling campaign” for the Indian region. The program encouraged mobile phone users to dispose of their used handsets and accessories, irrespective of the brand, at any of the 1,300 green recycling bins put up across the priority dealers and care centers. Nokia is also planning to launch an electronic waste management program.

The Department of Environment, Delhi government, has also decided to involve ragpickers in general waste management in the capital. These ragpickers will be trained, given uniforms, ID cards, and hired to clean waste. The department also intends to involve eco-clubs, now running in over 1,600 government and private schools in the Capital, in this initiative since it is these eco-clubs that will be interacting with ragpickers of that particular area.(28)

## Research on E-Waste Management

Many more environmental epidemiological studies are required to assess the present status of e-waste management system in India, to assess the e-waste quantities and exact amplitude of the problem in Indian cities, and to establish relationships with the informal recycling sectors. The valuable data will be generated by these studies that would help in drafting an action plan for e-waste management. India should start a surveillance system for diseases and health consequences of e-waste. The sustainability of e-waste management systems has to be ensured by improving the collection and recycling systems. It would be desirable to establish public-private partnerships in setting up buy-back or drop-off centers. Levying advance recycling fees is another approach to ensure waste management sustainability. To identify best e-waste management technologies across the globe and adopt them successfully can be key to a sustainable futuristic growth. The reduction of the hazardous substances in the electronic and electrical equipments and the promotion of use of their safer substitutes many countries have adopted the Restriction of Hazardous Substances (RoHS) Regulations in the manufacture of these items. More and more such less hazardous substitutes should be identified which can be used in electronic equipments.

## Conclusion

The hazardous nature of e-waste is one of the rapidly growing environmental problems of the world. The ever-increasing amount of e-waste associated with the lack of awareness and appropriate skill is deepening the problem. A large number of workers are involved in crude dismantling of these electronic items for their livelihood and their health is at risk; therefore, there is an urgent need to plan a preventive strategy in relation to health hazards of e-waste handling among these workers in India. Required information should be provided to these workers regarding safe handling of e-waste and personal protection. For e-waste management many technical solutions are available, but to be adopted in the management system, prerequisite conditions such as legislation, collection system, logistics, and manpower should be prepared. This may require operational research and evaluation studies.
